# A Self-Healing Optoacoustic Patch with High Damage Threshold and Conversion Efficiency for Biomedical Applications

**DOI:** 10.1007/s40820-024-01346-z

**Published:** 2024-02-19

**Authors:** Tao Zhang, Cheng-Hui Li, Wenbo Li, Zhen Wang, Zhongya Gu, Jiapu Li, Junru Yuan, Jun Ou-Yang, Xiaofei Yang, Benpeng Zhu

**Affiliations:** 1 School of Integrated Circuit, Wuhan National Laboratory for Optoelectronics, Huazhong University of Science and Technology, Wuhan, 430074 People’s Republic of China; 2 State Key Laboratory of Coordination Chemistry, Collaborative Innovation Center of Advanced Microstructures, School of Chemistry and Chemical Engineering, Nanjing University, Nanjing, 210093 People’s Republic of China; 3 National Institute of Dental and Craniofacial Research (NIDCR), National Institutes of Health (NIH), 35A Convent Drive, Bethesda, MD 20892 USA; 4 Department of Neurology, Tongji Hospital, Tongji Medical College, Huazhong University of Science and Technology, Wuhan, 430030 People’s Republic of China

**Keywords:** Optoacoustic, Self-healing PDMS, Acoustic flow, Thrombolytic, Wireless energy harvesting

## Abstract

Compared with traditional piezoelectric ultrasonic devices, optoacoustic devices have unique advantages such as a simple preparation process, anti-electromagnetic interference, and wireless long-distance power supply. However, current optoacoustic devices remain limited due to a low damage threshold and energy conversion efficiency, which seriously hinder their widespread applications. In this study, using a self-healing polydimethylsiloxane (PDMS, Fe-Hpdca-PDMS) and carbon nanotube composite, a flexible optoacoustic patch is developed, which possesses the self-healing capability at room temperature, and can even recover from damage induced by cutting or laser irradiation. Moreover, this patch can generate high-intensity ultrasound (> 25 MPa) without the focusing structure. The laser damage threshold is greater than 183.44 mJ cm^−2^, and the optoacoustic energy conversion efficiency reaches a major achievement at 10.66 × 10^−3^, compared with other carbon-based nanomaterials and PDMS composites. This patch is also been successfully examined in the application of acoustic flow, thrombolysis, and wireless energy harvesting. All findings in this study provides new insight into designing and fabricating of novel ultrasound devices for biomedical applications.

**Supplementary Information:**

The online version contains supplementary material available at 10.1007/s40820-024-01346-z.

## Highlights

- Based on Fe-Hpdca-PDMS and carbon nanotube composite, an optoacoustic patch is developed, which can recover from the damage induced by cutting or laser irradiation at room temperature.
- The patch has high laser damage threshold (183.44 mJ cm^−2^) and optoacoustic energy conversion efficiency (10.66×10^−3^).
- The patch has been successfully examined in acoustic flow, thrombolysis, and wireless energy harvesting, which may provide new insights into the field of the design and fabrication of novel ultrasound devices for biomedical applications.

**Supplementary Information:**

The online version contains supplementary material available at 10.1007/s40820-024-01346-z.

## Graphical abstract

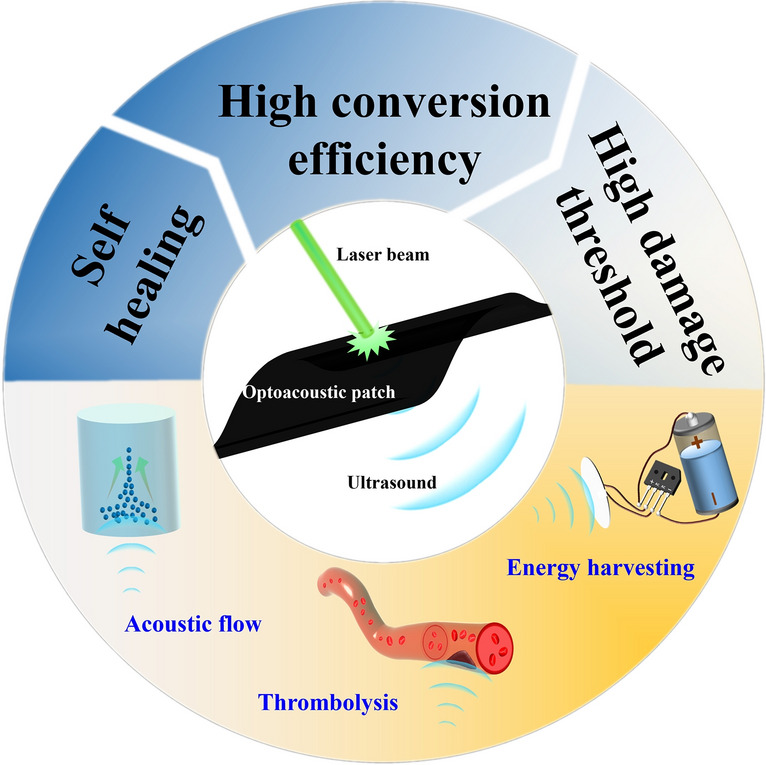

## Introduction

Ultrasonic devices are widely utilized in imaging [1], sonodynamic therapy [2], catalyst synthesis [3], drug delivery [4], neuromodulation [5–7], thrombolysis [8], and wireless energy harvesting [9–11]. In recent years, optoacoustic devices have attracted significant attention compared with traditional piezoelectric devices, due to their unique advantages such as simple preparation, electromagnetic compatibility, and high bandwidth [12–19]. In addition, inspired by the flexibility of piezoelectric acoustic patches [20, 21], flexible optoacoustic devices have become feasible, which are beneficial for portable applications. Because higher sound pressure output will always play a dominant role in biomedical treatment exploration and practical applications, such as microfluidics and energy harvesting, it is critical to develop high-power output optoacoustic devices.

Although high-intensity acoustic energy output can be obtained through the effective harvesting of laser-induced ultrasonic energy by adopting a focused physical structure, this approach requires complex manufacturing processes [18, 19]. Theoretically, the amplitude of the optoacoustic signal will elevate with increased laser energy. However, it instead drops when the laser energy density exceeds the laser damage threshold of the device [22]. In addition, the laser damage threshold of optoacoustic devices will limit the allowable laser energy input, affecting the upper level of acoustic energy output. Therefore, the maximum output of sound pressure can be increased by improving the damage threshold of optoacoustic devices. Alternatively, the development of optoacoustic composites with high energy conversion can serve as an important technical approach. Unfortunately, optoacoustic devices with high laser-induced damage threshold and optoacoustic energy conversion efficiency have rarely been reported. As shown in Table S1, self-healing materials have made some progress [23], and their related mechanisms mainly include hydrogen bonds [24], metal–ligand-interactions [25], Diels–Alder (DA) reactions [26], disulfide bonds [27], acylhydrazone bonds [28], π-π stacking [29], hydration [30], and anionic polymerization [31]. We previously reported on self-healing optoacoustic devices using urea-urethane elastomers. However, this device was based on a three-layer structure (self-healing nanocomposite/PDMS/glass) [18], and large-scale self-focusing physical structure was adopted to fulfill high sound pressure, which consequently posed a challenge for practical application.

**Fig. 1:**
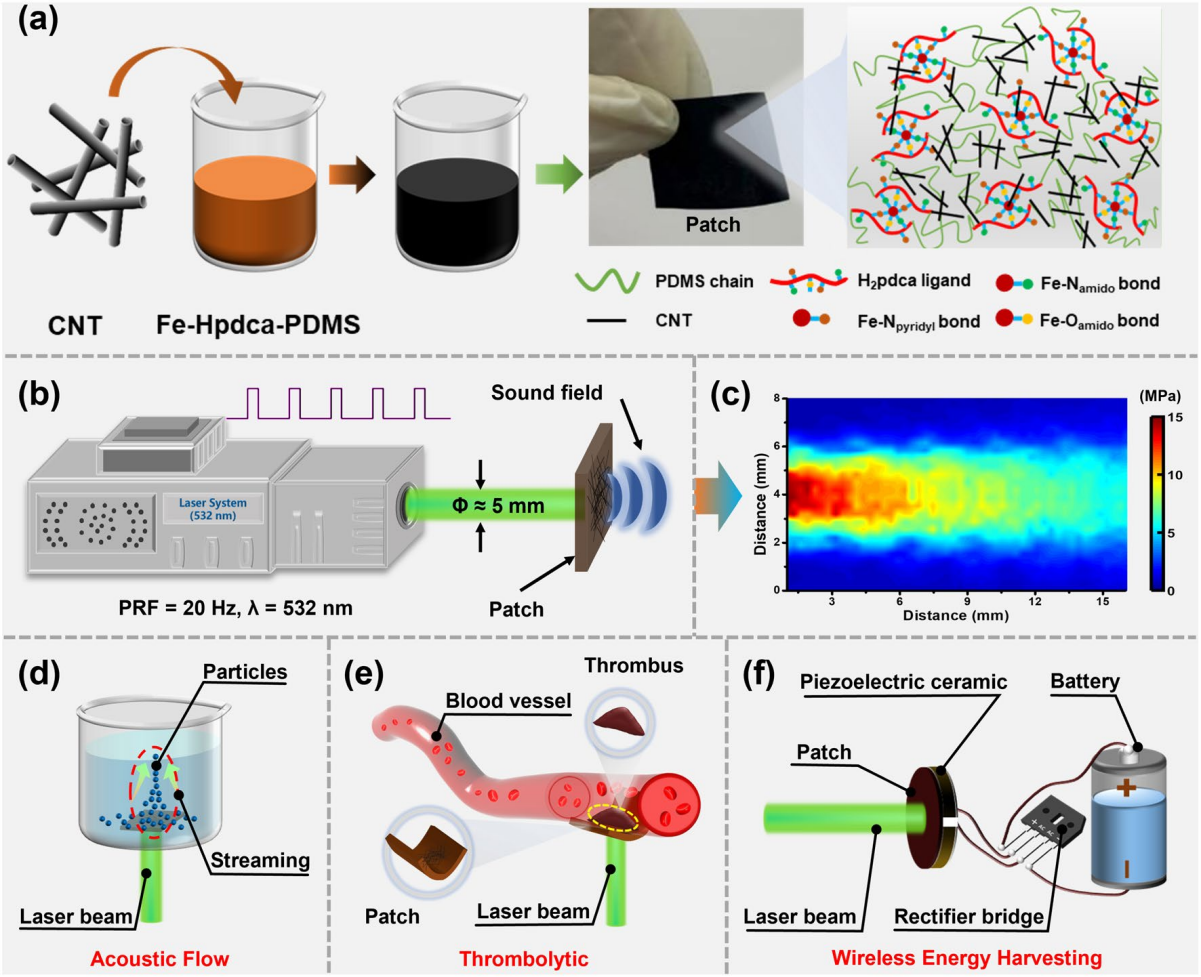
Design, fabrication, and application of the self-healing optoacoustic patch. **a** Device preparation method. **b** Optoacoustic patch under 532 nm laser excitation. **c** Acoustic field distribution of the patch. Applications in **d** acoustic flow, **e** thrombolytic, and **f** wireless energy harvesting

In this study, we created self-healing polydimethylsiloxane (PDMS, Fe-Hpdca-PDMS) with excellent self-healing properties at room temperature. As shown in Fig. 1a, an optoacoustic composite film was fabricated after mixing the light-absorbing material (carbon nanotube, CNT) and thermal expansion material (Fe-Hpdca-PDMS), which acted as a flexible patch. With pulsed laser irradiation (Fig. 1b), the patch immediately generated the effective output of acoustic energy (Fig. 1c). After exploring the influence of Fe-Hpdca-PDMS on the laser damage threshold, sound pressure output, and self-healing performance at different CNT concentrations, the patch could achieve a high damage threshold and energy conversion efficiency. In addition, the feasibility of practical patch applications was further examined by conducting acoustic flow (Fig. 1d), thrombolysis (Fig. 1e), and wireless energy harvesting experiments (Fig. 1f).

## Experimental Section

### Preparation of Self-healing Optoacoustic Patches

After chemical synthesis [32], self-healing PDMS (Fe-Hpdca-PDMS) materials were produced (Fig. S1). In order to fabricate self-healing optoacoustic patches with the different concentration of CNT (Length 20 μm and diameter 8 nm, Nanjing XFNANO Materials Tech Co.,Ltd, China), the mass ratio between the CNT and self-healing PDMS was kept at 4.0, 5.0, 6.7, 8.3, and 10.0 wt%. These two materials were then put into the tetrahydrofuran solution for mixing, and simultaneously transfer in a water bath (60 °C) for heating and concentrating. The spin coating (1000 r min^−1^, 60 s) was then carried out using a homogenizer (EZ4-S-PP, LEBO Science, China), and a composite film was obtained. After this film was heated and cured (80 °C, 2 h), the self-healing optoacoustic patch was fabricated finally.

### Device Performance Test

The thermal stability of the self-healing PDMS and ordinary PDMS was tested by a thermogravimetric analyzer (TGA8000, PerkinElmer, USA). Additionally, the glass transition temperature (*T*_g_) of the self-healing PDMS was measured by the differential scanning calorimeter (DSC) (DSC2500, TA Instruments, USA). The thermal expansion performance of self-healing PDMS was examined by a static thermal analyzer (TMA, TMA Q400EM, TA Instruments, USA), where the average linear expansion coefficient (*α*) and volume expansion coefficient ($\beta$) are expressed as following [33]:

1 $$\alpha = \frac{\Delta L}{{L}_{0}\times \Delta T}$$

2 $$\beta \approx 3\alpha$$

Among them, *∆L* denotes the length change of the measured object caused by the temperature change, ${L}_{0}$ is the initial value of the length of the measured object, and *∆T* represents the temperature change.

In the optoacoustic experiment, a 6 ns pulsed laser (λ = 532 nm) with a pulsed repetition frequency (PRF) of 20 Hz (Lapa-80, Beamtech Optronics, USA) was employed, and the initial aperture size of laser beam was about 5 mm. The laser-induced ultrasonic signal was detected by operating a fiber-optic hydrophone (diameter: 125 μm, FOH, Precision Acoustic, UK) with the sensitivity of 600 mV MPa^−1^, the frequency response range of 250 kHz ~ 50 MHz, and the sound pressure test range of 10 kPa ~ 15 MPa. Moreover, the hydrophone was moved by a 3D precision moving stage (H2-2206, ESM, CN). The resulting signal was displayed by a digital oscilloscope (TDS-2024B, Tektronix, USA). During the acoustic performance test in the self-healing process, the initial sound pressure of the device was first tested at 23 mJ pulse^−1^, followed by the irradiation of 50 mJ pulse^−1^ for 5 min. The sound pressure was then tested again and after 12 h at 23 mJ pulse^−1^. Meanwhile, the self-healing effect of the device was imaged using scanning electron microscopy (SEM, EVO-10, Zeiss, Germany).

### Acoustic Flow Experiment

Firstly, the self-healing optoacoustic patch was cut with a size of 10 mm × 10 mm, which was then attached to the bottom center of the beaker with 150 mL of deionized water added. In order to visualize the acoustic flow, 20 mL of ink was added to the water. Finally, the incident laser vertically irradiated to the center of the self-healing optoacoustic patch through the bottom of the beaker after reflecting by a 45° mirror. The process of optoacoustic flow formation was recorded by a camera (FDR-AX40, Sony, Japan).

### Thrombolytic Experiment

Firstly, the self-healing optoacoustic patch was cut with a size of 15 mm × 20 mm, and then sticked to the top of the Petri dish. Normal saline was poured to immerse the optoacoustic patch. Then, the blood clots were placed on top of the optoacoustic patch, and microbubbles (Sonazoid, GE Healthcare AS, Norway) with the concentration of 4.3 × 10^7^ MBs mL^−1^ were injected to the pump at a speed of 0.5 mL min^−1^. Finally, the incident laser vertically irradiated to the center of the self-healing optoacoustic patch (~ 15 MPa, 20 Hz) through the bottom of the Petri dish after reflecting by a 45° mirror, and the thrombolysis experiment process was recorded by a camera.

### Wireless Energy Harvesting Experiment

Firstly, the self-healing optoacoustic patch was cut into a circular block with a diameter of 10 mm, which was then adhered to one side of the PZT ceramic using uncured PDMS (1 MHz, Table S2). The laser was directly applied vertically to the optoacoustic patch, and the output electrical signal of the piezoelectric ceramic was observed using an oscilloscope. Regarding charging test of 47 μF capacitor, the average charging power ($\overline{P}$) is calculated by the following formula [9]:

3 $$\overline{P}= \frac{{C}_{s}{{V}_{0}}^{2}}{2T}$$

In which ${C}_{s}$ is the capacitance, *T* is the charging time, and ${V}_{0}$ is the effective output voltage. In the experiment of laser driven piezoelectric energy harvesting, one side of PZT ceramics with the same parameters was used to receive the laser drive. The same experiment was tested in deionized water.

## Results and Discussion

### Fabrication and Characterization of the Self-healing Optoacoustic Patch

Common optoacoustic devices are typically composed of light-absorbing and thermal expansion materials. Currently, light-absorbing materials consisting of carbon-based nanomaterials, such as carbon black (CB) [34], carbon nanotube (CNT) [35, 36], candle soot nanoparticles (CSNPs) [37], carbon nanofiber (CNF) [38], and reduced graphene oxide (rGO) [39], have been extensively investigated. These nanomaterials were found to outperform other candidates in absorbing light energy and promoting heat transfer [40], which serve as key factors in improving optoacoustic conversion efficiency and obtaining high-amplitude sound pressure signals. Among carbon-based nanomaterials, CNT have excellent light absorption characteristics, as well as good thermal properties. In addition, CNT have much higher thermal diffusivity (~ 5.3 × 10^−5^ m^2^ s^−1^) than CSNPs [37]. As a result, CNT are considered an ideal option for light-absorbing materials. Hence, in this study, CNT were employed as the light-absorbing material.

PDMS is mainly used in optoacoustic devices as a thermal expansion material. However, the Grüneisen coefficient of PDMS will attenuate with increased temperature [41], decreasing the optoacoustic conversion efficiency and degrading its performance. Therefore, to fabricate optoacoustic devices with a high laser damage threshold and optoacoustic conversion efficiency, other thermal expandable materials with higher thermal expansion coefficients and thermal stability were explored. Recently, it was reported that a coordination complex cross-linked polymer chain network (Fe-Hpdca-PDMS) exhibited not only self-healing characteristics at room temperature but also high strength and tensile properties [32].

**Fig. 2:**
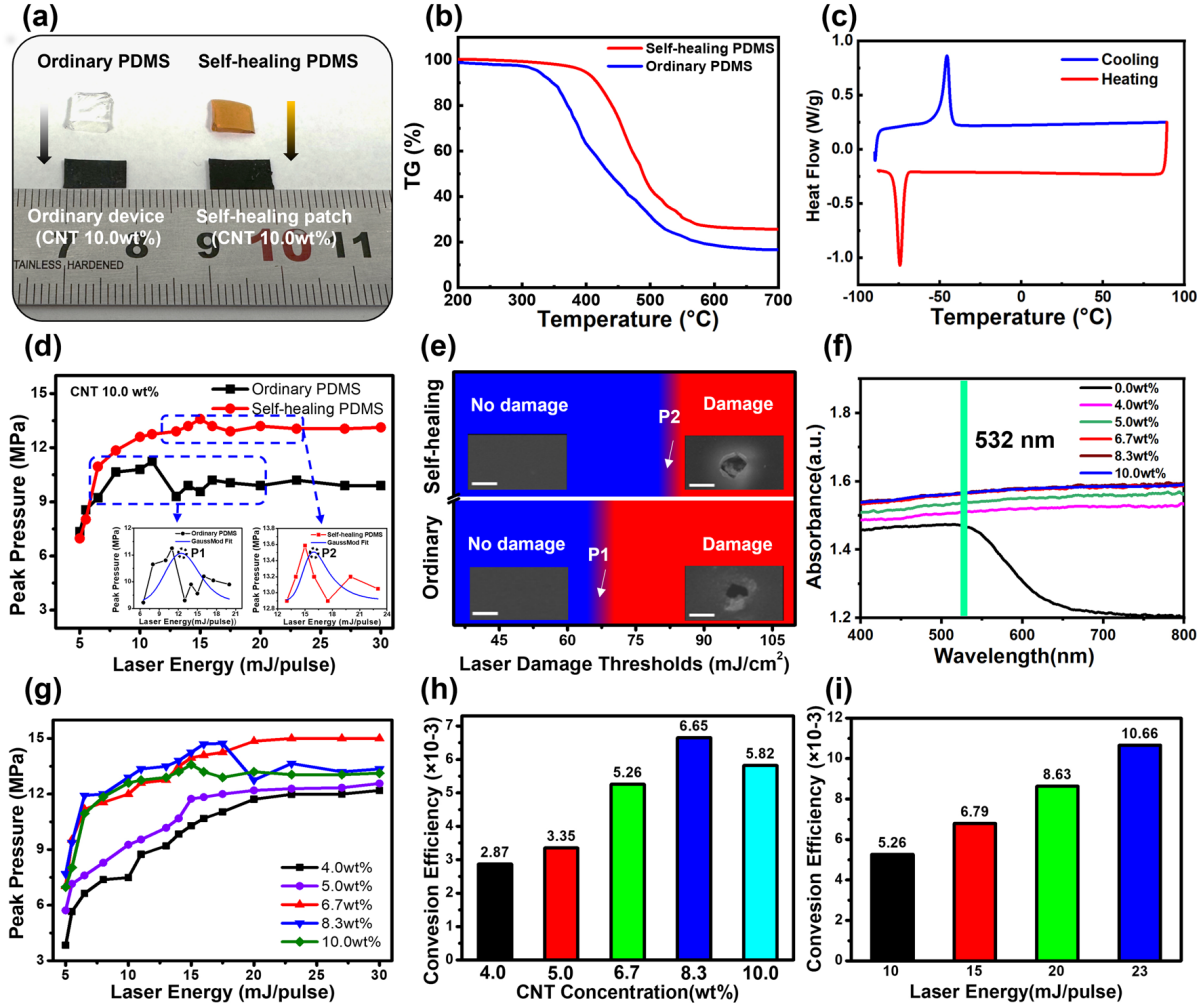
Acoustic performance test of the self-healing optoacoustic patch. **a** Size/shape of the ordinary PDMS and self-healing PDMS (10.0 wt% CNT). **b** Comparison of thermogravimetric analysis between the self-healing PDMS and ordinary PDMS. **c** DSC test of the self-healing PDMS. **d** Peak sound pressure output of the optoacoustic device based on the self-healing PDMS and ordinary PDMS under different laser intensities. Points P1 and P2 represent the peak points of fitted curves. **e** Heat map of laser damage thresholds between the self-healing patch and ordinary device. The left patches are at the initial state, and the right are post-damage, on a scale bar of 50 μm. **f** Laser absorption in different CNT concentrations (0.0, 4.0, 5.0, 6.7, 8.3, and 10.0 wt%). **g** Changes of peak sound pressure at different excitation laser energies in various CNT concentrations. **h** Optoacoustic energy conversion efficiency in different CNT concentrations at 10 mJ pulse^−1^. **i** Optoacoustic energy conversion efficiency with 6.7 wt% of CNT at different excitation laser intensities

The self-healing PDMS (Fe-Hpdca-PDMS) material was prepared as shown in Fig. S1. To characterize the thermal performance of the self-healing PDMS, devices based on ordinary PDMS and self-healing PDMS were compared (Fig. 2a). As shown in Fig. 2b, the thermal stability testing indicated that the mass of self-healing PDMS was basically unchanged before 300 °C, indicating no water of crystallization in the polymer chain. Between 300 and 400 °C, the quality slightly decreased. Starting from 400 °C, it dropped sharply, which was probably because the polymer chain skeleton started to decompose as the temperature increased. By contrast, the quality of ordinary PDMS started to decline sharply as early as ~ 300 °C, which indicated that the pyrolysis temperature of self-healing PDMS was higher than ordinary PDMS. These results indicated that self-healing PDMS had better thermal stability, which laid the foundation for the excellent damage threshold and broad practical applications of patches based on self-healing PDMS. Figure 2c demonstrates that a distinct endothermic peak (− 74.32 °C) and exothermic peak (− 45.54 °C) were observed in the glass transition temperature (*T*_g_) of the self-healing PDMS, illustrating that a considerably low *T*_g_ point existed in self-healing PDMS. The *T*_g_ point of the self-healing PDMS was lower than room temperature, which was beneficial for polymer flow, and promoted self-healing at room temperature. According to the thermal expansion performance test results (Fig. S2), the thermal expansion coefficient of the self-healing PDMS was calculated as ~ 10.35 × 10^−3^ K^−1^, which was higher than ordinary PDMS (9.2 × 10^−3^ K^−1^) [42]. Moreover, when the self-healing PDMS was cut into two parts (Fig. S3a), it could be cured after 24 h at room temperature (Fig. S3b). Certainly, the mechanism of such self-healing phenomenon came from the metal–ligand-interactions of Fe-Hpdca-PDMS [32].

The above findings elucidated that self-healing PDMS could better retain thermal stability, with a higher thermal expansion coefficient and a unique self-healing function, compared with ordinary PDMS. According to theoretical derivation (Note S1), the higher the coefficient of thermal expansion, the higher the optoacoustic conversion efficiency. Therefore, the better the thermal stability, the higher the laser energy that the optoacoustic device could withstand, ultimately leading to high sound pressure output. Therefore, the laser damage threshold of the transducer could be improved by utilizing self-healing PDMS as a thermal expansion material, further facilitating the design of high-performance self-healing optoacoustic devices.

Following the optoacoustic device fabrication process (Experimental Section), we manufactured a flexible self-healing optoacoustic patch (Fig. S4). Figure 2d shows the performance comparison of optoacoustic devices fabricated by self-healing PDMS and ordinary PDMS, in which the CNT concentration was maintained at 10.0 wt%. Under the same energy laser irradiation (> 6 mJ pulse^−1^), the optoacoustic device based on self-healing PDMS could generate higher peak sound pressure, indicating that self-healing PDMS was beneficial for optoacoustic conversion efficiency. In addition, the P1 and P2 peak points in the fitting curve indicated that the output sound pressure of the patches was not enhanced with increased laser energy, indicating the critical damage threshold point. As shown in Fig. 2e, the optoacoustic device based on self-healing PDMS also maintained a higher laser damage threshold (81.2 mJ cm^−2^), due to the high thermal stability of the self-healing PDMS. In addition, as shown in Fig. S5, in terms of center frequency and bandwidth performance, the self-healing PDMS-based patch (8.0 MHz, 125.8% at − 6 dB) was consistent with the ordinary PDMS-based patch (8.0 MHz, 122.8% at − 6 dB).

To explore the effect of CNT concentration on the acoustic pressure, laser damage threshold, and self-healing performance, various self-healing optoacoustic patches were fabricated with CNT concentrations of 0.0, 4.0, 5.0, 6.7, 8.3, and 10.0 wt%. Figure 2f demonstrates that near 532 nm, the self-healing optoacoustic patches with various CNT concentrations had a high light absorption capacity, and the values increased with increasing CNT concentration. When the CNT concentration was higher than 6.7 wt%, the absorbance reached saturation and no longer increased. As shown in Fig. 2g, the peak acoustic pressure increased along with increasing CNT concentration before the device was damaged by the laser. This indicated that the higher the CNT concentration, the higher the absorbance, and the more sufficient contact between the CNTs and PDMS. As a result, heat generated by the CNTs was more easily transferred to the PDMS. In addition, the patch (6.7 wt% CNT) had a good laser damage threshold of 183.44 mJ cm^−2^ (Table S3). Notably, although a device with a CNT concentration of 6.7 wt% already produced an optoacoustic signal with a peak sound pressure (15 MPa), when the laser energy was 20 mJ pulse^−1^, it was limited by the hydrophone test range (peak sound pressure of 1 kPa to 15 MPa). After conducting the experiments (Note S2), we found that the maximum output sound pressure was greater than 25 MPa. As shown in Fig. 2h, when the laser energy was held at 10 mJ pulse^−1^, the optoacoustic energy conversion efficiencies of the devices with five CNT concentrations were 2.87 × 10^−3^, 3.35 × 10^−3^, 5.26 × 10^−3^, 6.65 × 10^−3^, and 5.82 × 10^−3^. When the laser energy was 23 mJ pulse^−1^, the optoacoustic conversion efficiency of the patch with a CNT concentration of 6.7 wt% reached 10.66 × 10^−3^ (Fig. 2i), achieving the highest reported value for carbon nanomaterial-based optoacoustic devices (Table S4).

When the CNT concentration was low (less than 6.7 wt%), the damage track on the surface of the device almost disappeared (Fig. S6). Alternatively, when the concentration was increased (greater than 6.7 wt%), the self-healing ability gradually became weak, and the damage was only partially restored, and distinct marks remained. Therefore, when the CNT concentration was low, many binding sites was present between the internal metal ions and the ligands in the self-healing PDMS, ultimately making it easier to reform the metal–ligand coordination bonds. Conversely, when the CNT concentration was too high, the number of internal dynamic metal–ligand coordination bonds was reduced [32]. In addition, the conductivity was verified by the conductivity test of the self-healing patch (Fig. S7).

### Self-healing Performance of the Optoacoustic Patch

**Fig. 3:**
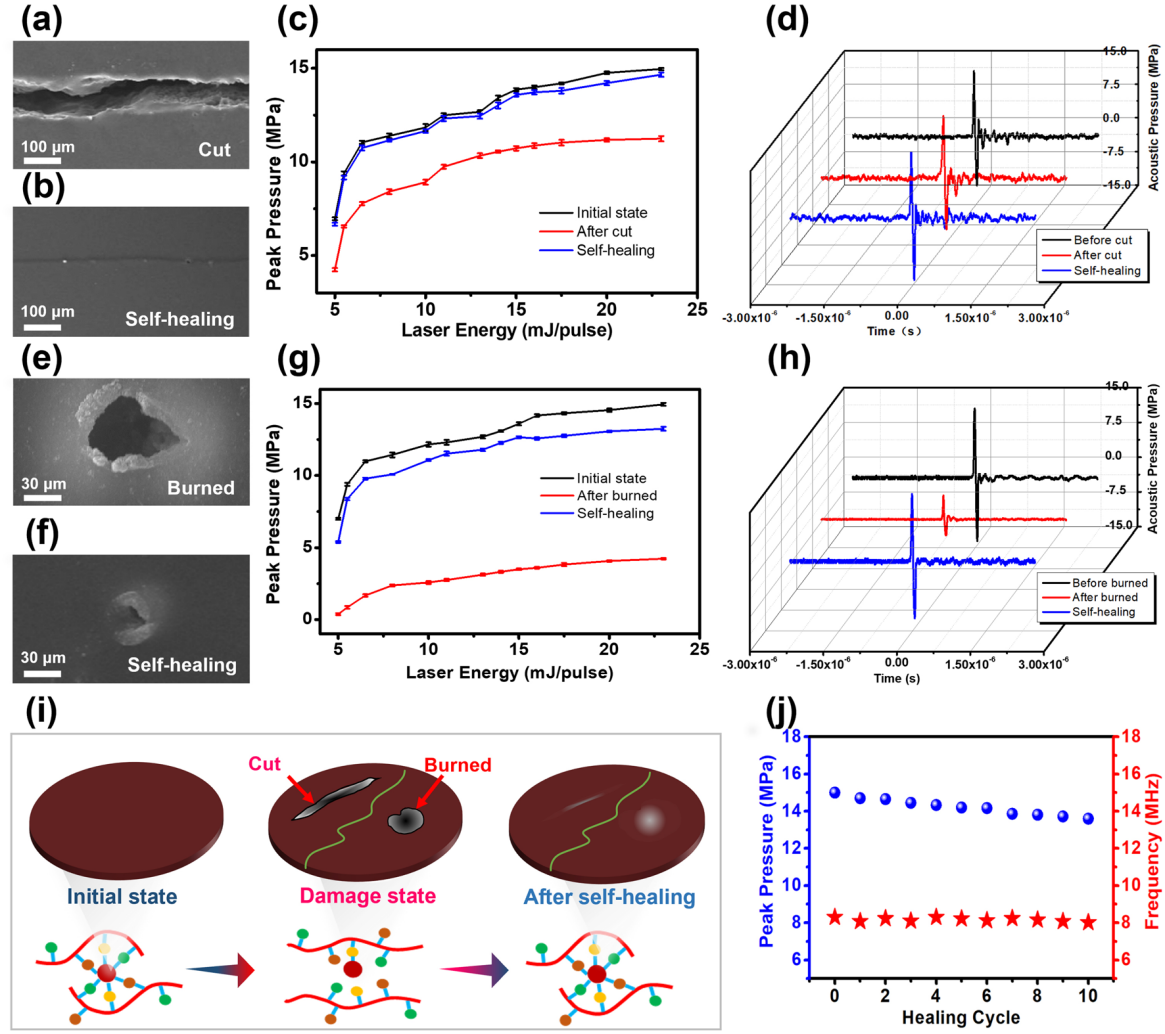
Self-healing performance examination of the self-healing optoacoustic patch. The damage track after **a** cut and **b** self-healing. **c** Ultrasonic peak pressure and **d** output waveform before cut (initial state), after cut, and after self-healing at different input laser energy. Each experiment is repeated four times. The damage track after **e** burning and **f** self-healing. **g** Ultrasonic peak pressure and **h** output waveform before burning (initial state), after burning, and after self-healing. Each experiment is repeated four times. **i** Schematic diagram of self-healing process. **j** Peak sound pressure and center frequency after several times of cut/self-healing

As shown in Fig. 3a, the surface morphology of the self-healing patch (CNT of 6.7 wt%) was tested after cutting. After 12 h of self-healing at room temperature, the cut-damaged surface of the patch recovered (Fig. 3b). The sound pressure test (Fig. 3c) demonstrated that physical damage (cut) seriously influenced the output sound pressure of the transducer, and the maximum output sound pressure (11.2 MPa) was only 74.7% of the initial state (15 MPa) at a laser energy of 23 mJ pulse^−1^. However, after self-healing, the maximum output sound pressure (14.7 MPa) of the patch could be restored to 98.0% at the same laser energy. Furthermore, no obvious alternations of the output signal waveform from the optoacoustic patch were observed after physical damage (Fig. 3d). When high-intensity laser radiated to the patch, damage caused by thermal breakdown will be occur. Due to thermal inhomogeneity, the damage region was smaller than the size of the laser beam (Fig. S8). As shown in Fig. 3e, obvious damage was observed (burned) on the surface of the device after laser irradiation with an intensity of 50 mJ pulse^−1^ for 5 min, and the device can achieve a certain degree of self-healing after 12 h at room temperature (Fig. 3f). The sound pressure test results illustrated that the output sound pressure of the burning-damaged optoacoustic patch dropped to 4.2 MPa, reaching 13.2 MPa (88.0% of the initial state) after self-healing (Fig. 3g) at a laser energy of 23 mJ pulse^−1^. No obvious alternations were detected with respect to the waveform of the output signal (Fig. 3h).

Both physical and thermal damage caused fracture of the dynamic metal ligand in the self-healing PDMS. Specifically, if the damage strength was far from the irreversible limit of the material, the metal ions in the damaged area remained near the ligand through strong interactions, enabling rapid bond formation, followed by self-healing (Fig. 3i). In addition, the self-healing patch maintained relatively stable performance after repeated cutting damage (Fig. 3j). However, the performance of the patch started to deteriorate after multiple thermal damage, and it almost failed after 20 times with an intensity of 50 mJ pulse^−1^ for 5 min (Fig. S9). Both cutting damage and thermal breakdown (burned), the center frequency of patches kept at 8.0 MHz (Figs. S10 and S11). Compared with cutting damage, the thermal breakdown process was accompanied by physical damage and irreversible chemical damage. The irreversible chemical damage would accumulate with increasing number of the damage, and further weaken the patch performance by hindering the formation of the metal ligand coordinate covalent bonds.

We summarized the related parameters of several laser-induced ultrasound devices based on composite structures comprising carbon-based nanomaterials and PDMS (Table S4). To date, only poly (urea urethane)-CNT/PDMS laser-induced ultrasound devices based on a layered structure of self-healing nanocomposite/PDMS/glass substrates have been reported with self-healing characteristics. Although a self-healing effect was achieved, the center frequency and optoacoustic conversion efficiency were compromised in a previous study. In addition, based on the above results, in order to maintain long-term high-performance application of the self-healing patch, the laser incident intensity needs to be reduced and maintains within the damage threshold (183.44 mJ cm^−2^).

### Acoustic Flow Application of the Self-healing Optoacoustic Patch

Microfluidics technology has been widely used in drug delivery, DNA synthesis, cell screening, and other fields due to its virtue of small size, portability, and other characteristics in microfluidics equipment [43, 44]. As the basis of microfluidics equipment, the micropump serves as the power source of microfluidic transport.

**Fig. 4:**
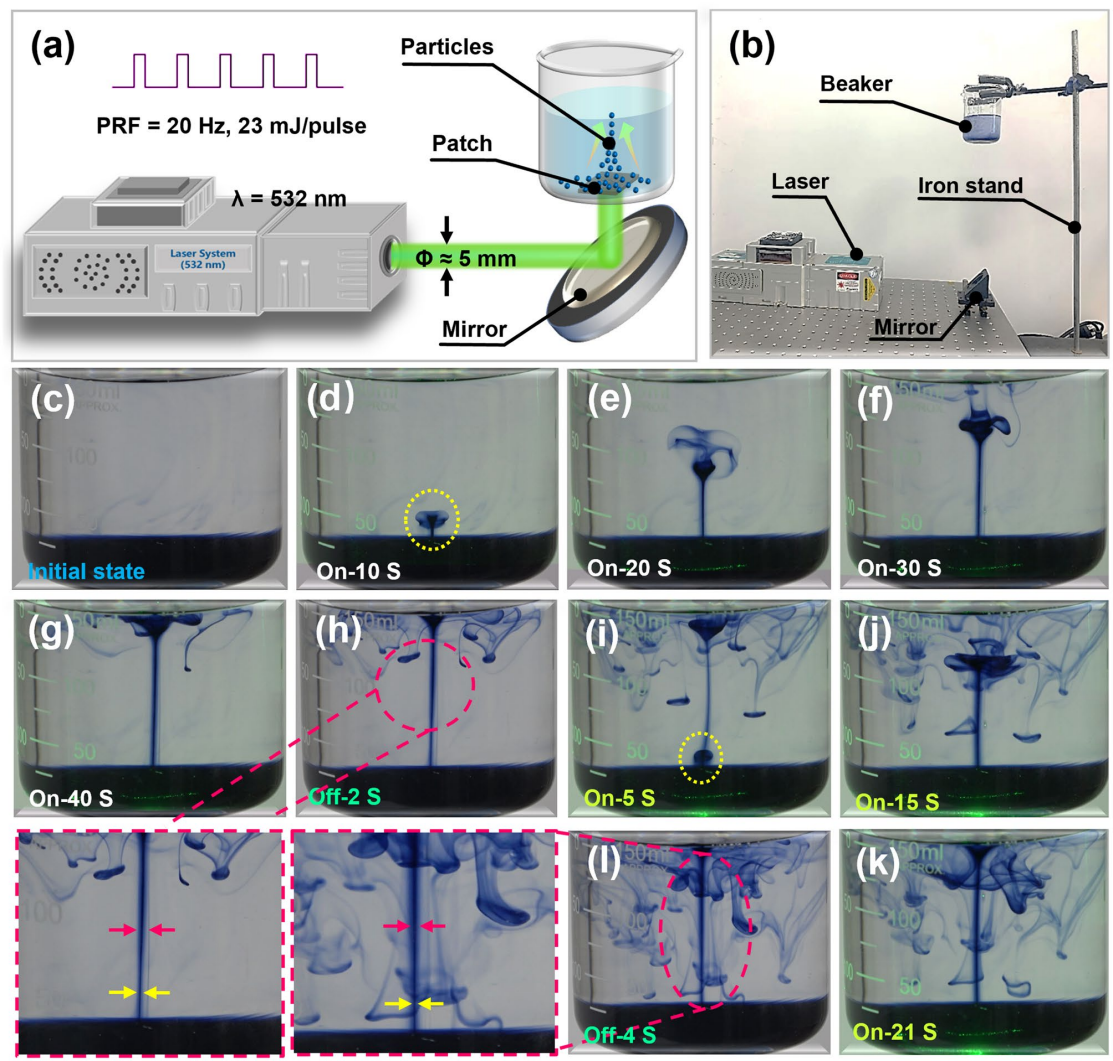
Acoustic flow experiment of the self-healing optoacoustic patch. **a** Schematic diagram of experimental design. **b** Setup of the experiment. **c** Initial state. **d**-**g** Ink state change at 10^th^, 20^th^, 30^th^, and 40^th^ s after laser irradiation. **h** Ink state after turning off the laser for 2 s. **i**-**k** Ink state change at 5^th^, 15^th^, and 21^th^ s after turning on the laser again. **l** Ink state after turning off the laser again at 4^th^ s

Because self-healing optoacoustic patches possess high laser damage threshold features and high-intensity sound pressure output, without focusing on structures examined by previous measurements, they may serve as an optoacoustic micropump. Therefore, we conducted an optoacoustic flow experiment utilizing self-healing optoacoustic patches (Fig. 4a, b). Finally, the process of optoacoustic flow formation was captured. Notably, when the laser was off at the beginning, blue ink deposited at the bottom of the beaker (initial state) (Fig. 4c). After the laser (23 mJ pulse^−1^, and 20 Hz) was initiated for 10 s, a mushroom cloud-shaped ink mass appeared above the patch (Fig. 4d). With further laser irradiation, the ink continued to rise (Fig. 4e, f), and the diameter of the acoustic flow of the blue ink was approximately 2.0 mm due to its sound field. After 40 s of application, the ink increased to the liquid level (Fig. 4g), followed by colliding with the liquid surface. After tracing the ink trajectory, it was evident that stable sound flow existed in the middle of the beaker, in the laser incidence direction. Therefore, when the sound field generated by the optoacoustic patch propagated in water, the spatial gradient of the sound field changed due to viscous attenuation, causing the medium to flow in the sound beam along the propagation direction, forming a stable Eckart sound flow field [45]. Because the size of the carbon black particles in the ink was at the nanoscale [46], and the acoustic radiation force acting on the carbon black particles was much smaller than the drag force caused by the acoustic flow field, the drag force played a dominant role in driving the particles [47]. To verify reproducibility, we proceeded using similar procedures as described above. Specifically, after switching off the laser, the diameter of the ink’s trajectory diminished (Fig. 4h). When the laser was off, the acoustic flow field disappeared and carbon black particles were no longer affected by the drag force. Only a few particles continued to move due to inertia. After turning the laser on for 5 s, a mushroom cloud-shaped ink mass reemerged (Fig. 4i), which continued to rise during laser irradiation (Fig. 4j), finally reaching the top again (Fig. 4k). When the laser was powered off again, the diameter of the ink’s trajectory decreased again, as shown in Fig. 4l (Movie S1).

To validate that the movement of the ink was influenced by the acoustic flow field rather than the laser, a comparative experiment was conducted, where the self-healing optoacoustic patch was removed. The results demonstrated that although the carbon black particles of the ink moved under laser irradiation, its trajectory was chaotic and became disordered (Fig. S12 and Movie S2). Two main reasons were involved. First, carbon black particles absorbed the energy of the laser and generated heat, causing local liquid expansion and flow. Second, carbon black particles mixed in the liquid could generate optoacoustic signals under laser irradiation. However, the intensity was weak and the movement lacked directionality. All these findings indicated that stable sound flow stemmed only from the self-healing optoacoustic patch.

### Thrombolytic Application of the Self-healing Optoacoustic Patch

**Fig. 5:**
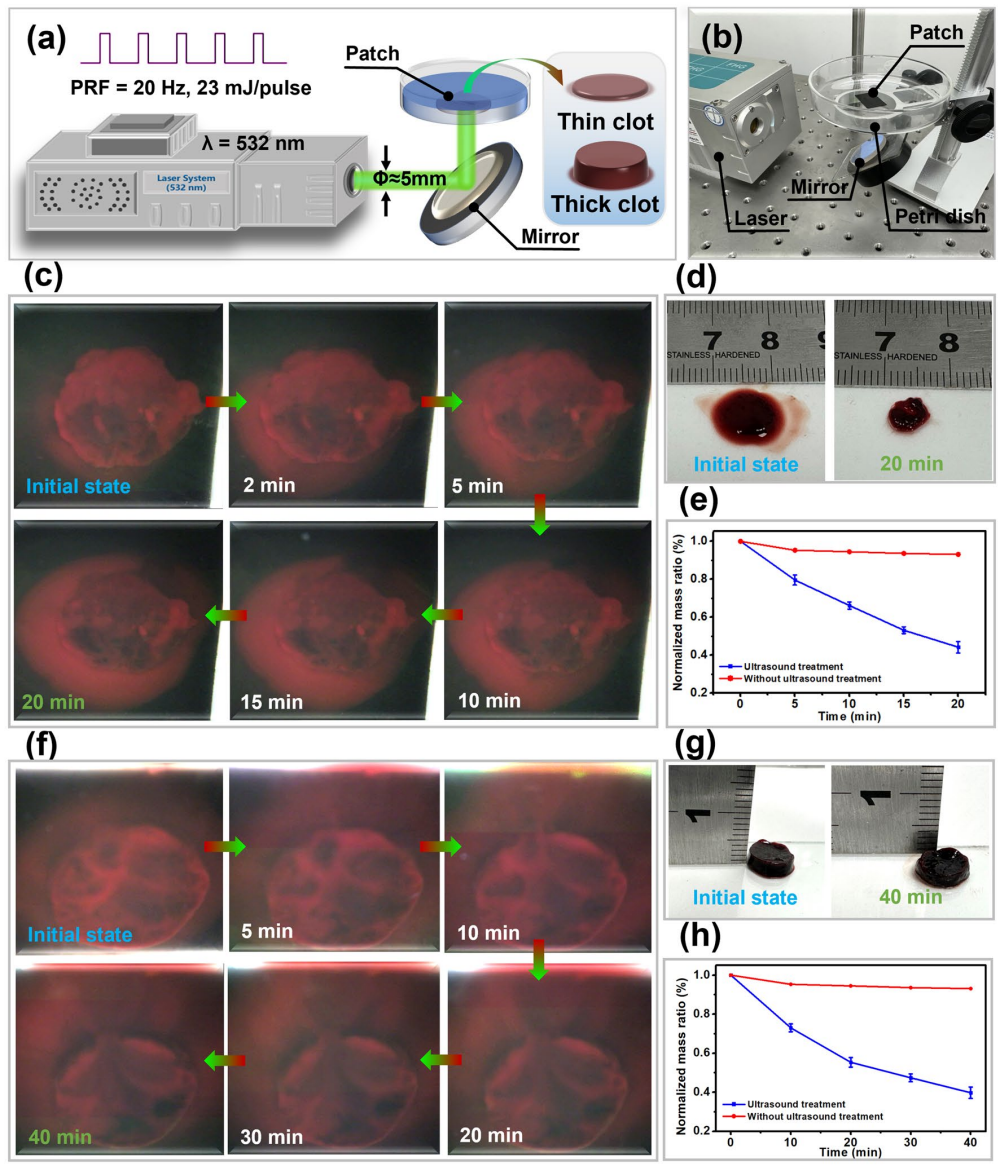
Thrombolytic experiment of self-healing optoacoustic patch. **a** Schematic diagram of experimental design. **b** Setup of the experiment. **c** State changes of thin blood clots at the initial moment, 2^th^, 5^th^, 10^th^, 15^th^, and 20^th^ minute of optoacoustic action. **d** Thin blood clot in its initial state and after 20 min of ultrasound treatment. **e** Normalized mass change curve of thin blood clots, in which the control group (red line) without ultrasound treatment and the experimental group (green line) with ultrasound treatment (n = 4). **f** Changes in the state of thick blood clots at the initial moment, 5^th^, 10^th^, 20^th^, 30^th^, and 40^th^ minute of optoacoustic application. **g** Thick blood clot in its initial state and after 40 mins of ultrasound treatment. **h** Normalized mass change curve of thick blood clots, in which the control group (red line) without ultrasound treatment and the experimental group (green line) with ultrasound treatment (n = 4)

In the field of biomedicine, ultrasound-accelerated thrombolysis has recently emerged as a promising technique for treating thrombosis by transmitting sound waves to blood clots [48–50]. Currently, common ultrasound thrombolysis equipment is based on focusing the ultrasound transducer structure, which requires accurate focus control during the treatment process. The optoacoustic flow experiment indicated that the self-healing optoacoustic patch still maintained a high sound intensity output without a focusing structure. Therefore, we examined the feasibility of thrombolysis induced by the optoacoustic patch. An experiment was designed to visualize the thrombolysis process, as shown in Fig. 5a, b. Specifically, a mirror was used to reflect the laser to an optoacoustic patch attached to a transparent Petri dish with a blood clot sample placed on the patch. Because the thrombolysis time and ultrasound intensity could be reduced by ultrasound microbubbles, as investigated by many studies [51], a laser intensity of 23 mJ pulse^−1^ was used, and ultrasound microbubbles were injected to attenuate the damage caused by optoacoustic patches during long-term action of the high-intensity laser. In addition, two types of thrombus models were implemented, namely, a thin blood clot and a thick blood clot, to investigate the thrombolytic effect.

As shown in Fig. 5c, two minutes after applying the laser, the blood stains around the thin blood clot became considerably enlarged, and the dark-colored areas became more prominent. After 5, 10, 15, and 20 min, the blood stains around the blood clot spread further (Movie S3). After 20 min, the blood clot was removed, and the size was smaller than in the initial state (diameter > 10.0 mm) (Fig. 5d). Compared with no ultrasound treatment, the mass of thin blood clots was obviously reduced after ultrasound treatment, but the reduction rate gradually slowed down over time (Fig. 5e). Furthermore, after 5, 10, 15, 20, 30, and 40 min of optoacoustic action, the thick blood stain gradually expanded and became prominent in the dark area in the middle (Fig. 5f and Movie S4). After 40 min of application, the blood clot was removed, and the thickness decreased to about 1.5 mm (Fig. 5g). Multiple pits were also observed on the surface, and the diameter was reduced (Fig. S13). Although the mass change pattern of the thick blood clots was the same as those of thin blood clots, much longer decay time was observed in thick blood clots (Fig. 5h). The main reason was that thin blood clots were easily penetrated and the contact surface with microbubbles increased. All results illustrated the feasibility of thrombolytic application by utilizing the optoacoustic patch.

### Wireless Energy Harvesting of the Self-healing Optoacoustic Patch

**Fig. 6:**
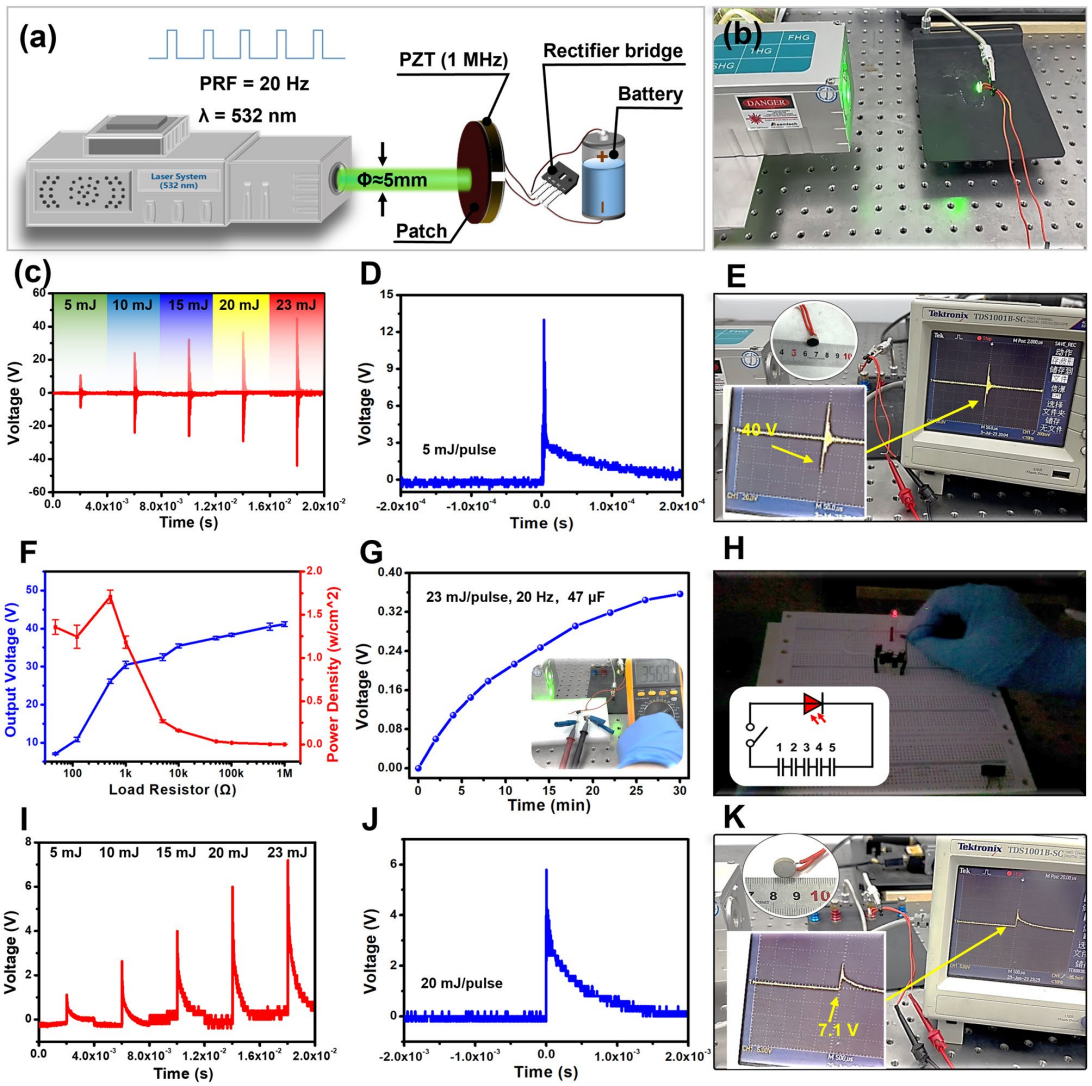
Wireless energy harvesting of the self-healing optoacoustic patch. **a** Schematic diagram of experimental design. **b** Setup of the experiment. **c** Open circuit voltage of wireless energy harvesting devices based on self-healing optoacoustic patches at different laser intensities. **d** Rectification output at 5 mJ pulse^−1^ laser intensity. **e** Oscilloscope readout (40 V) of wireless energy harvesting device. **f** Output voltage and output power of a wireless energy harvesting device based on self-healing optoacoustic patches at the load impedances of 47, 120, 510, 1, 5, 10, 51, 100, 510 kΩ, and 1 MΩ. Each experiment is repeated four times. **g** Charging voltage change of the 47 μF capacitor. The inset denotes the saturation voltage. **h** Setup of the lighting experiment. **i** Open circuit voltage of pure laser driven piezoelectric ceramics. **j** Open circuit voltage amplification of a piezoelectric ceramic driven at 20 mJ pulse^−1^ laser intensity. **k** Oscilloscope readout (7.1 V) of a pure laser driven piezoelectric ceramic

In recent years, wireless energy harvesting technology based on ultrasound has gained significant attention in wireless energy supply applications for both intelligent electronic and neural regulatory devices [52–54]. Due to the excellent self-healing ability and high sound pressure output of the self-healing optoacoustic patch, we conducted wireless energy harvesting application experiments, as shown in Fig. 6a, b. PZT (Table S2 and Fig. S14) piezoelectric ceramics were used as collectors for optoacoustic energy. The results of the open-circuit voltage test indicated that the output voltage of the piezoelectric collector was gradually enhanced as the laser intensity increased (Fig. 6c). After rectification, a good output waveform was also maintained (Fig. 6d). When the laser intensity was 23 mJ pulse^−1^, the output voltage was also higher than 40 V and maintained a reliable signal-to-noise ratio (SNR = 11.69 dB), which was feasible for wireless energy harvesting, as shown in Fig. 6e (Fig. S15 and Movie S5). Furthermore, the load testing results indicated that the instantaneous output power density of the optoacoustic wireless energy harvesting device reached 1.7 W cm^−2^ when the input laser was 23 mJ pulse^−1^ and the load was 530 Ω (Fig. 6f). However, the actual charging capacity was greatly limited because the pulse repetition rate of the pulsed laser was only 20 Hz. Moreover, after charging a 47 μF capacitor for 30 min, the saturation voltage increased to 0.357 V (Fig. 6g) and the average charging power was 1.7 nW. By charging five 47 μF capacitors in a series circuit, a red LED could be illuminated, as shown in Fig. 6h (Movie S6), fulfilling the application requirements of electronic devices. Hence, the charging ability could be remarkably promoted by employing high pulse repetition rate lasers.

Pure lasers can realize wireless energy harvesting when acting on piezoelectric ceramics [55, 56]. To explore the main wireless energy harvesting source of this optoacoustic patch, a purely laser-driven piezoelectric energy harvesting experiment was performed. Driven by different laser intensities, the voltage gradually increased (Fig. 6i), but was merely 7.1 V under the action of 23 mJ pulse^−1^, as shown in Fig. 6k (Movie S7). In addition, the output voltage across the piezoelectric ceramic remained monophasic without the patch (Fig. 6j). This phenomenon was due to the laser pressure signal, which was monophasic, and was significantly different from the formed optoacoustic biphasic signal (Fig. S16). These findings also indicated that the energy output by the piezoelectric ceramics was mainly derived from the acoustic energy of the patch in this study. We found that the device still maintained superior electrical output performance when the device was placed in water (Fig. S17 and Movie S8), which further expanded the application field of wireless energy harvesting based on the optoacoustic patch.

## Conclusions

In summary, we developed a self-healing optoacoustic patch by combining CNT with self-healing PDMS (Fe-Hpdca-PDMS) materials, which possessed a self-healing capability at room temperature, and could recover from damage induced by cutting or laser irradiation. Moreover, a high laser damage threshold (183.44 mJ cm^−2^) and high energy conversion efficiency (10.66 × 10^−3^) were obtained. In contrast to other optoacoustic devices, the patch still achieved a high-intensity sound pressure output (> 25 MPa) in a non-focusing structure. Based on the self-healing optoacoustic patch, the application of optoacoustic flow, thrombolysis, and wireless energy harvesting was realized. Compared with traditional piezoelectric ultrasonic transducers, the patch possessed the characteristics of no electrical interconnection, a simple structure, and self-healing properties. The development of this optoacoustic patch provided a new approach to designing and fabricating novel ultrasound devices for biomedical applications.

## Supplementary Information

Below is the link to the electronic supplementary material.

Supplementary file1 (MP4 34310 KB)

Supplementary file2 (MP4 38508 KB)

Supplementary file3 (MP4 38242 KB)

Supplementary file4 (MP4 22731 KB)

Supplementary file5 (MP4 9744 KB)

Supplementary file6 (MP4 12153 KB)

Supplementary file7 (MP4 7077 KB)

Supplementary file8 (MP4 9832 KB)

Supplementary file9 (PDF 1606 KB)
