# Supplementary material for: A Self-Healing Optoacoustic Patch with High Damage Threshold and Conversion Efficiency for Biomedical Applications
Source: Nanomicro Lett. 2024 Feb 19;16:122. doi: 10.1007/s40820-024-01346-z (PMC10876513; doi:10.1007/s40820-024-01346-z)
Supplement: Supplementary file 9 — Supplementary file9 (PDF 1606 KB) [file 40820_2024_1346_MOESM9_ESM.pdf]

Supporting Information for

A Self-Healing Optoacoustic Patch with High Damage Threshold and Conversion Efficiency for Biomedical Applications

Tao Zhang[1, #], Cheng-Hui Li[2, #], Wenbo Li[1], Zhen Wang[3], Zhongya Gu[4], Jiapu Li[1], Junru Yuan[1], Jun Ou-Yang[1], Xiaofei Yang[1], and Benpeng Zhu[1, *]

[1] School of Integrated Circuit, Wuhan National Laboratory for Optoelectronics, Huazhong University of Science and Technology, Wuhan 430074, P. R. China

[2] State Key Laboratory of Coordination Chemistry, School of Chemistry and Chemical Engineering, Collaborative Innovation Center of Advanced Microstructures, Nanjing University, Nanjing 210093, P. R. China

[3] National Institute of Dental and Craniofacial Research (NIDCR), National Institutes of Health (NIH), 35A Convent Drive, Bethesda, MD 20892, USA

[4] Department of Neurology, Tongji Hospital, Tongji Medical College, Huazhong University of Science and Technology, Wuhan 430030, P. R. China

[#]Tao Zhang and Cheng-Hui Li contributed equally to this work.

* Corresponding author. E-mail: benpengzhu@hust.edu.cn (B. Zhu)

Supplementary Figures and Tables

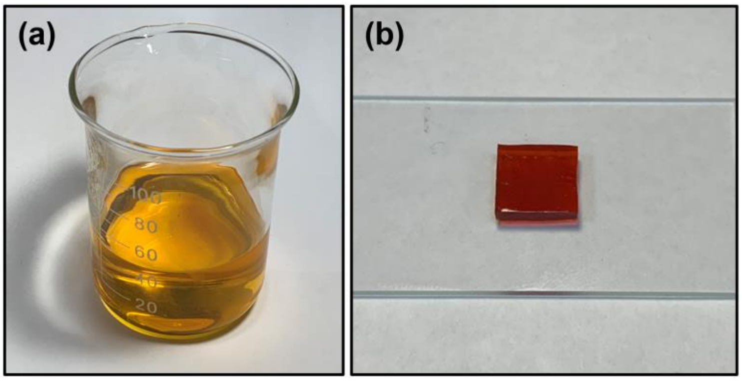

Fig. S1 Self-healing PDMS. **a** Before concentration. **b** After concentration and curing

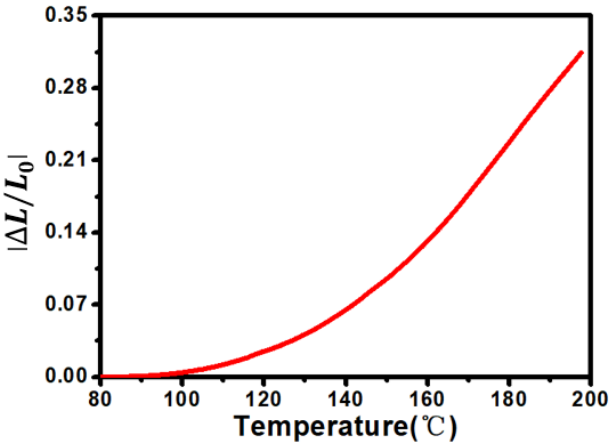

Fig. S2 TMA test of the self-healing PDMS

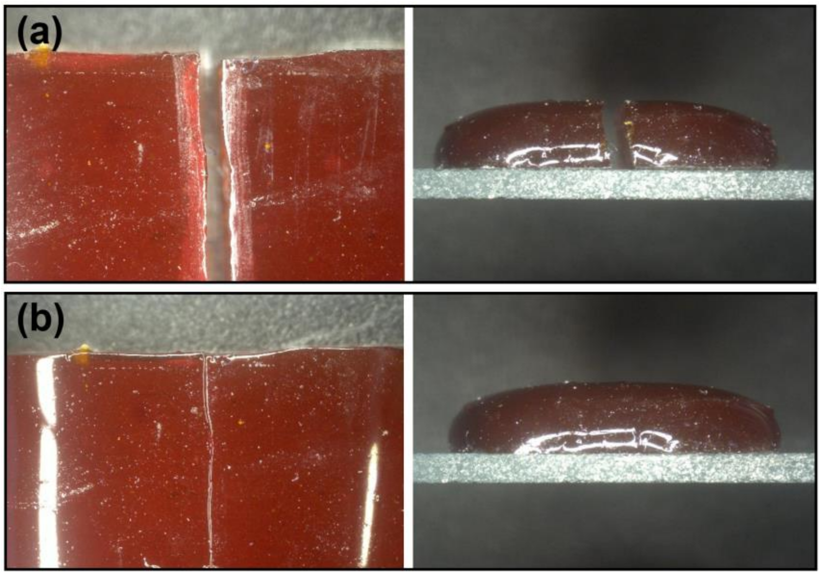

Fig. S3 The process of the self-healing PDMS at room temperature. **a** Before self-healing. **b** After self-healing

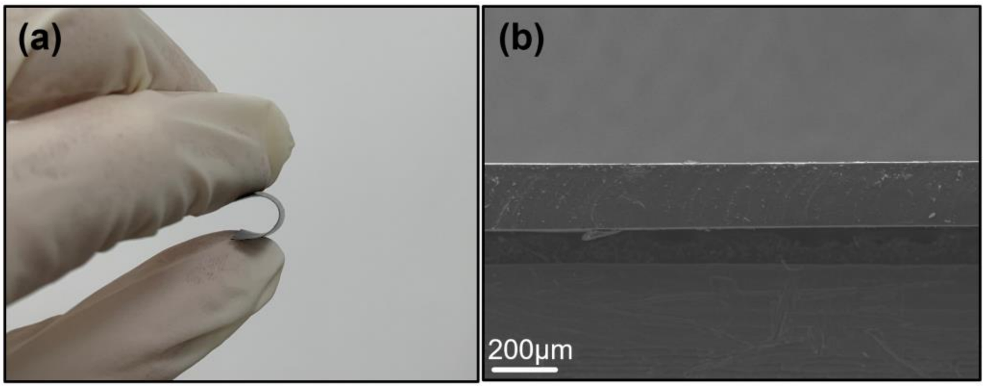

Fig. S4 The self-healing optoacoustic patch. **a** Side view. **b** SEM image

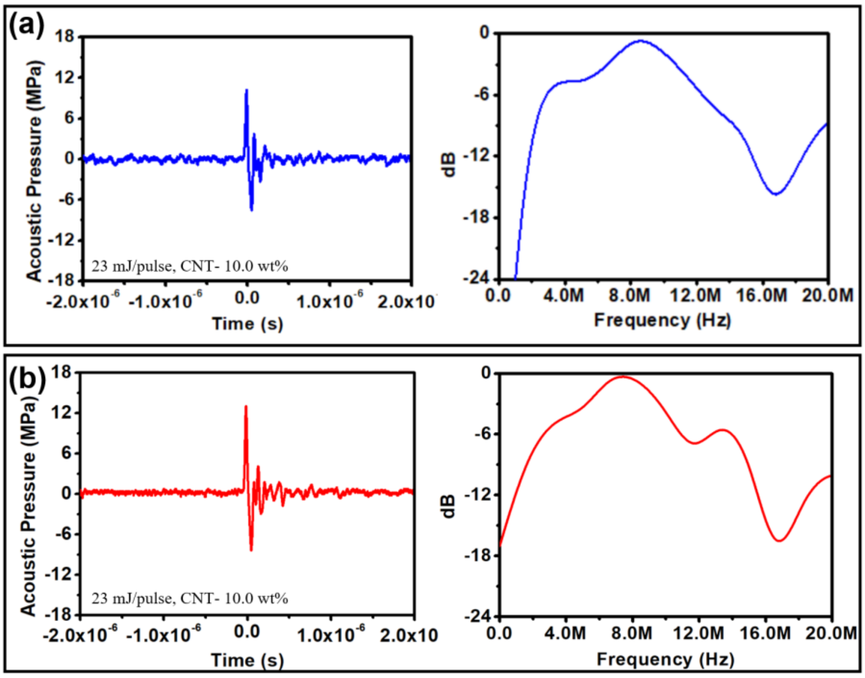

Fig. S5 Waveform and spectrogram of the ordinary PDMS based optoacoustic patch (**a**) and self-healing PDMS based optoacoustic patch (**b**)

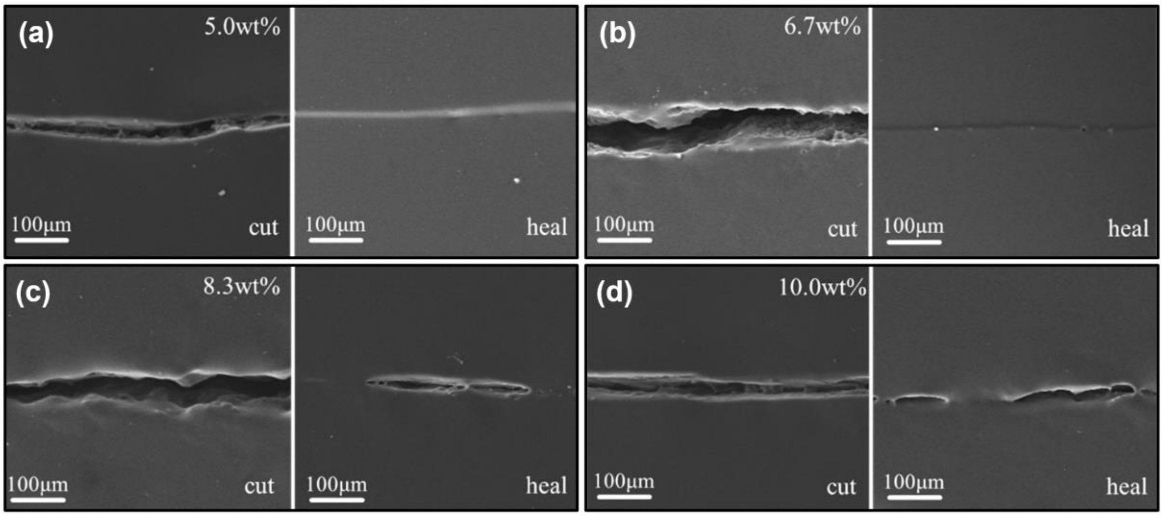

Fig. S6 Comparison of self-healing performance of self-healing optoacoustic patches with different CNT concentrations. **a** 5.0 wt%. **b** 6.7 wt%. **c** 8.3wt%. **d** 10.0 wt%

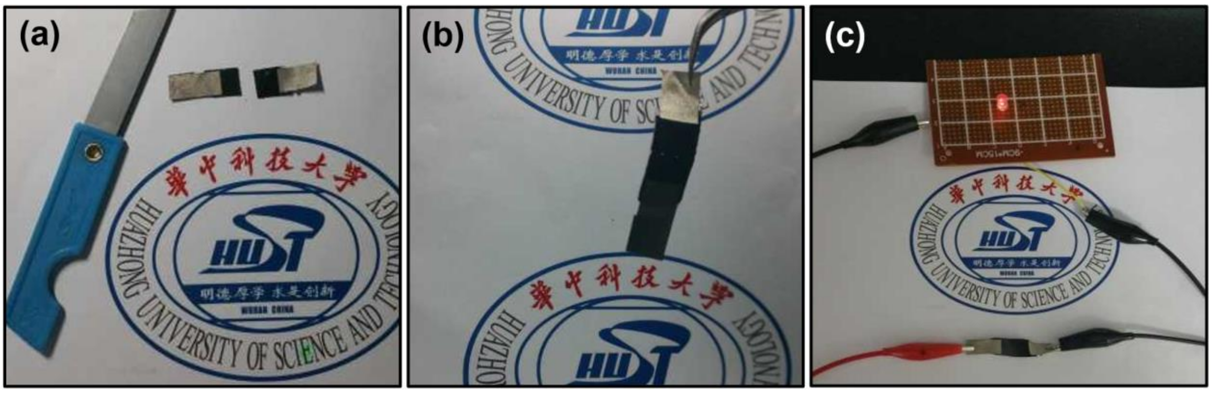

Fig. S7 The self-healing performance of the self-healing optoacoustic patch. **a** Before cutting. **b** After self-healing. **c** Electrical conduction by the self-healing optoacoustic patch

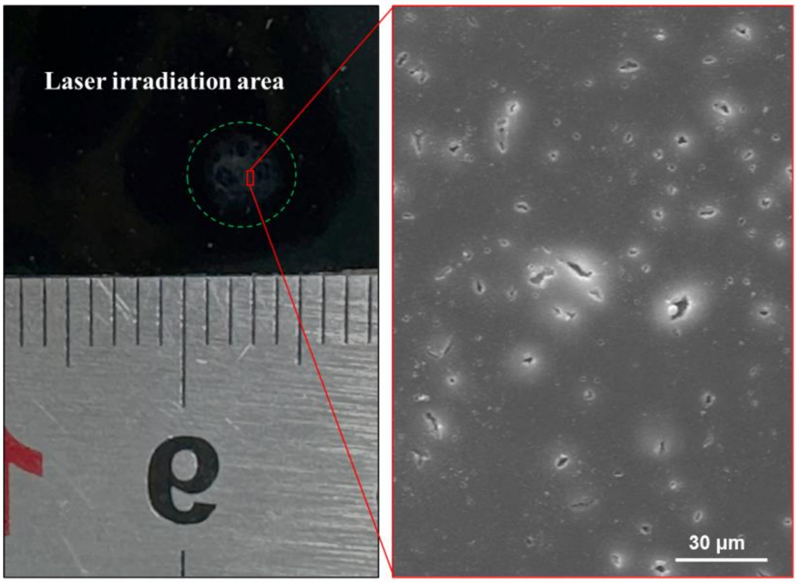

Fig. S8 The photo of the laser irradiated area and SEM image of local thermal damage area at 50 mJ/pulse for 5 min

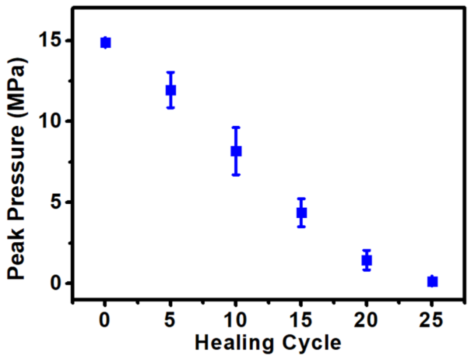

Fig. S9 Peak sound pressure after several times of burning/self-healing with an intensity of 50 mJ/pulse for 5 min (n = 4)

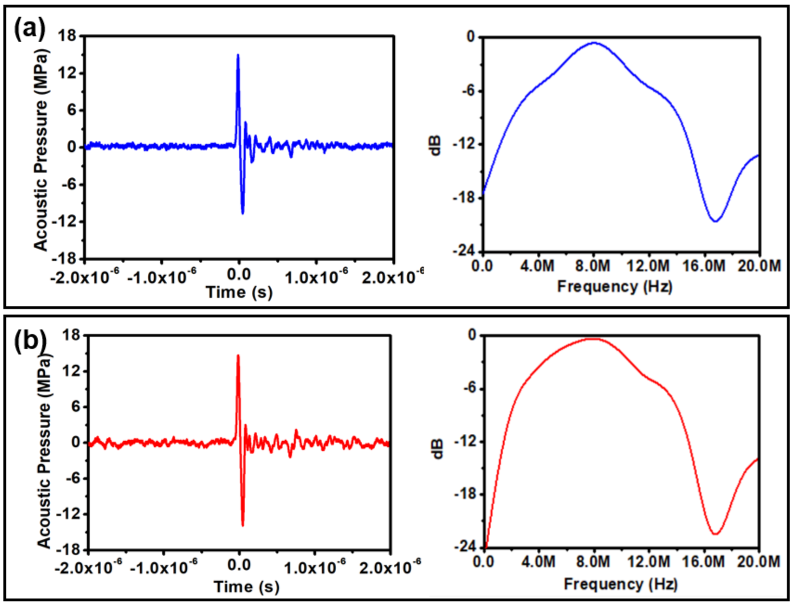

Fig. S10 Waveform and spectrogram of the self-healing optoacoustic patch after cut. Waveform and spectrogram in **a** the initial state and **b** after self-healing

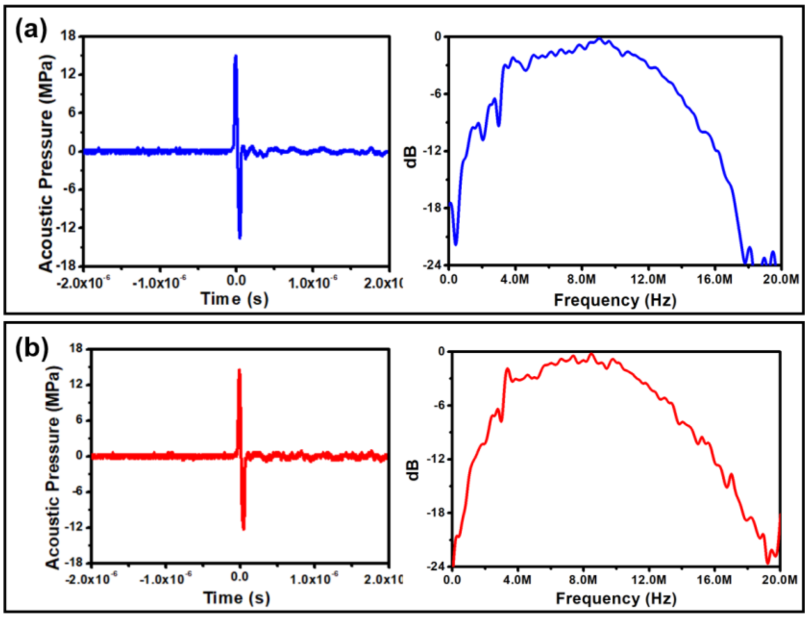

Fig. S11 Waveform and spectrogram of the self-healing optoacoustic patch after burning. Waveform and spectrogram in **a** the initial state and **b** after self-healing

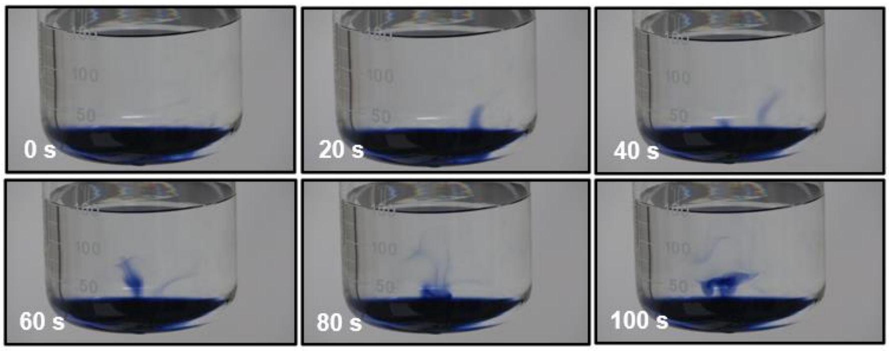

Fig. S12 Acoustic flow without the optoacoustic patch

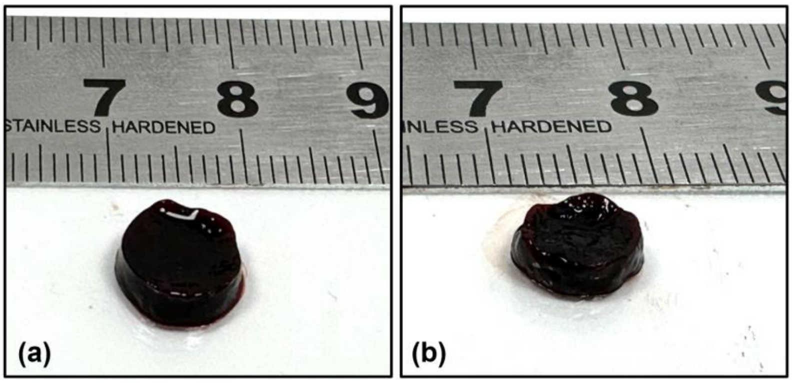

Fig. S13 Size/shape changes of thick blood clots. **a** Before optoacoustic treatment. **b** After optoacoustic treatment

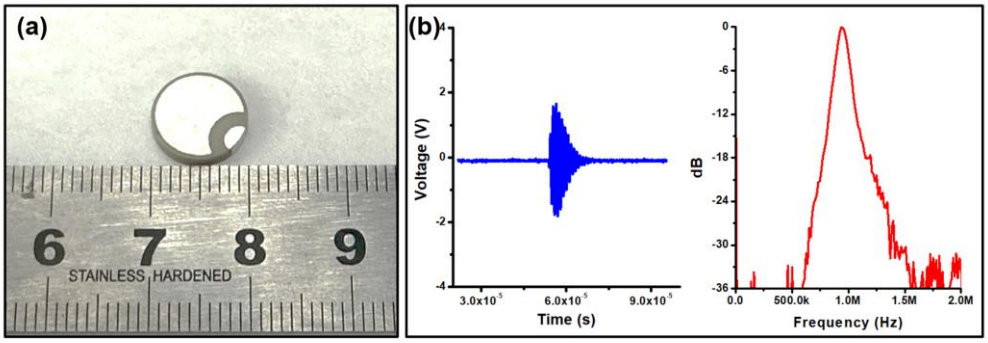

Fig. S14 PZT piezoelectric ceramic. **a** The size/shape of ceramic. **b** Echo and spectrum diagram

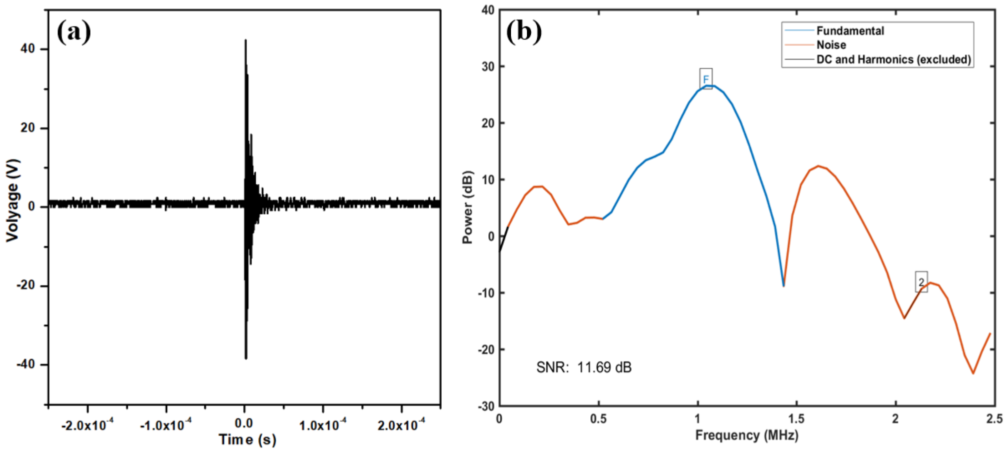

Fig. S15 Signals received by the wireless energy harvesting devices. **a** Time domain diagram of the received signal (40 V). **b** The power spectrum diagram (SNR = 11.69 dB)

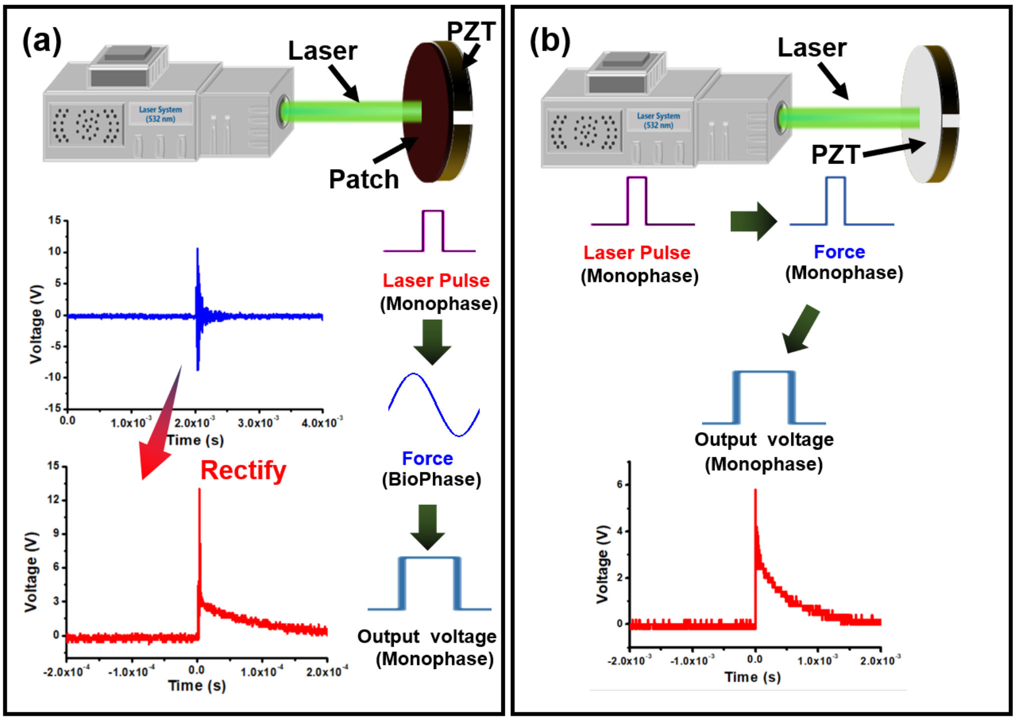

Fig. S16 The comparison of outputs of energy collection based on the optoacoustic patch and laser energy collection. **a** With and **b** Without the optoacoustic patch

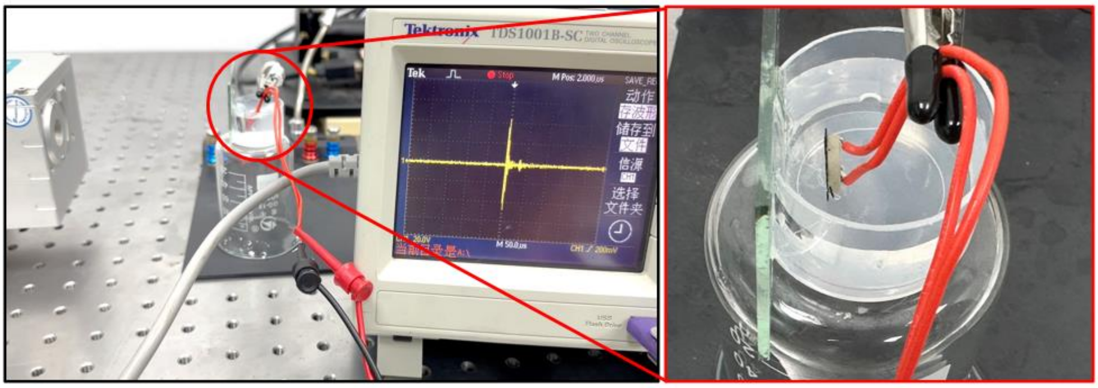

Fig. S17 The optoacoustic patch for wireless energy harvesting in water

Table S1 Damage types of self-healing materials and their self-healing mechanisms

Materials	Damages	Mechanisms	Refs.
PDMS-MPU	Physical cutting	Hydrogen bonds	[S1]
Metal complexes	Physical cutting	Metal-ligand-interactions	[S2]
Furan/Maleimide	Physical cutting	Diels-Alder (DA) reactions	[S3]
Synthesized PUSA	Physical cutting	Disulfide bonds	[S4]
Hydrogels with PNIPAM copolymers and OSA	Physical cutting	Acyl hydrazone bonds	[S5]
PVA-Py with CNTs	Physical cutting	π-π stacking	[S6]
Cement pastes with carbon nanomaterials	Thermal damage	Hydration	[S7]
EMI-modified epoxy with microcapsules	Electrical damage	Anionic polymerization	[S8]

Table S2 Parameters of the PZT piezoelectric ceramic

Parameter	Value
**Piezoelectric coefficient d_{33}**	410 pC/N
**Electromechanical coupling coefficient k_{33}**	64%
**Thickness *h***	2 mm
**Diameter Ø**	10 mm
**Density *ρ***	7600 kg/m^3
**Center frequency *f***	1 MHz

Table S3 The comparison of laser damage thresholds of optoacoustic devices

Optoacoustic devices		Laser Damage Threshold（mJ/cm^2）
Ref. [S9]		22.93
Ref. [S10]		81
Ref. [S11]		108.6
Ref. [S12]		178.34
This work	CNT of 4.0 wt%	> 183.44
	CNT of 5.0 wt%	> 183.44
	CNT of 6.7 wt%	> 183.44
	CNT of 8.3 wt%	101.91
	CNT of 10.0 wt%	81.2

Table S4 The comparison of optoacoustic devices based on carbon-based nanomaterials-PDMS composites

Laser absorbing material/thermal expansion material	Substrate	Laser energy density (mJ/cm^2)	Center frequency (MHz)	Maximum output positive/negative sound pressure (MPa)	Optoacoustic conversion efficiency (×10^{-3})
CNF/PDMS [S13]	Glass	3.71	3	+12.15/-1	~1.66
CSNP/PDMS [S9]	Glass	3.57	10	+4.8/-1	4.41
CNT/PDMS [S12]	No	178.34	7.8	+15/-2	2.99
Poly(urea-urethane)-CNT/PDMS [S14]	Glass	203.82	2.5	+15/-8	~3.52
This work	No	117.20	8	+15/-13.55	10.66

The optoacoustic conversion efficiency (η) is calculated by the following formula [S15]:

$$\eta = \frac{1}{F}\int_{-\infty}^{+\infty}\frac{p^2(t)}{\rho c}dt \qquad \text{(S1)}$$

In which, F is input laser energy density, p is instantaneous sound pressure, ρ is density ($p_{water} = 1000\ kg/m^3$), and c is the speed of sound ($c_{water} = 1480\ m/s$).

Note S1 Theory of Optoacoustic Conversion Efficiency

The mechanism of the laser-induced ultrasonic effect is that the optoacoustic composite material (light-absorbing material/thermal expansion material) firstly absorbs the energy of the short-pulse laser, then generating periodic thermal expansion and contraction, thereby emitting ultrasonic waves. It includes a complex energy conversion process: from light energy to thermal energy, and then to mechanical energy (sound waves) [S16]. Moreover, the generation of optoacoustic signals needs to meet thermal and stress constraints: the duration of the laser pulse should be shorter than the thermal relaxation time to generate a large temperature gradient [S16]; the pulse width should be shorter than the acoustic relaxation time [S17]. Therefore, nanosecond pulsed lasers are usually utilized to irradiate optoacoustic composites to generate strong optoacoustic signals. The optoacoustic pressure amplitude (P) obtained by optoacoustic composites is expressed as [S18, S19]:

$$P = \Gamma \cdot A \cdot \frac{F}{L} \qquad \text{(S2)}$$

where $\Gamma = \beta c^2/C_p$ is the Grüneisen coefficient, L is defined as the characteristic length of the absorption region (m), A is the light absorption rate (dimensionless), F is the laser energy density (J/m^2), β is the volume thermal expansion coefficient (K^{-1}), c is the speed of sound (m/s), C_p is the heat capacity (J/K).

The characteristic length of the absorption region (L = max ($c\tau_l$, $1/\alpha$)) depends on the maximum of the thickness of the absorption region or the penetration depth of the incident light, where τ_l is the pulse laser width (ns) and α is the absorption coefficient (cm^{-1}), formula (S1) can be further expressed as:

$$P = \begin{cases} \dfrac{\beta c^2}{C_p} \cdot A \cdot \dfrac{F}{c\tau_l} & c\tau_l \gg 1/\alpha \\ \dfrac{\beta c^2}{C_p} \cdot A \cdot \dfrac{F}{\frac{1}{\alpha}} & c\tau_l \ll 1/\alpha \end{cases} \qquad \text{(S3)}$$

The above formula (S3) shows that the optoacoustic pressure amplitude gained by the pulsed laser is proportional to the material's own characteristics and the laser irradiation conditions, which provides guidance for the study of high-performance optoacoustic composite materials. In order to obtain high-amplitude optoacoustic signals, optoacoustic composite materials are required to have not only a high light absorption coefficient, but also a large thermal expansion coefficient, high wave velocity, and low heat capacity. In addition, it is obvious that a large input laser energy density is also the key to generate high-amplitude optoacoustic signals.

The optoacoustic conversion efficiency is the ratio of the output sound pressure energy to the incident laser energy, which is an important standard to measure the performance of the optoacoustic transducer. For high optoacoustic conversion efficiency, the thickness of the light-absorbing layer should be much smaller than the acoustic propagation length within the duration of the laser pulse, as long as it does not significantly affect its light-absorbing properties. Therefore, the generation of optoacoustic signals in thin light-absorbing layers should conform to the long-pulse mechanism ($c\tau_l \gg 1/\alpha$). Moreover, the generation of optoacoustic signals in optoacoustic composites is mostly classified as a long-pulse mechanism, and the optoacoustic conversion efficiency can be expressed as the above formula (S1).

Under the long-pulse mechanism, since the time distribution of optoacoustic pulses is equal to that of Gaussian laser pulses, P(t) can be expressed by the duration of laser pulses (τ_l) [S20]:

$$p(t) = p_0 exp\left(-ln2\frac{t^2}{{\tau_l}^2}\right) \quad \text{(S4)}$$

$$\int_{-\infty}^{+\infty} p^2(t)\,dt = 0.75 \cdot {p_0}^2\tau_l \quad \text{(S5)}$$

Then combine Eqs. (S3) and (S5) to determine the optoacoustic conversion efficiency under long pulse conditions [S20]:

$$\eta = 0.75 \cdot A^2 \cdot (\frac{\beta}{C_p})^2 \cdot \frac{cF}{\rho\tau_l} \quad \text{(S6)}$$

Therefore, higher light absorption rate (*A*), thermal expansion coefficient (*β*), and input laser energy density (*F*), lead to higher optoacoustic conversion efficiency (*η*).

Note S2 Sound Pressure Approximation Test

In this study, the device with a CNT concentration of 6.7 wt% has already produced an optoacoustic signal with a peak sound pressure close to 15 MPa when the laser energy is 20 mJ/pulse. However, it is limited due to the hydrophone testing range (peak sound pressure 1 kPa~15 MPa). Although the laser energy continues increasing, the actual sound pressure cannot be measured (beyond the test range). Hence, we design an approximate experiment with the following method:

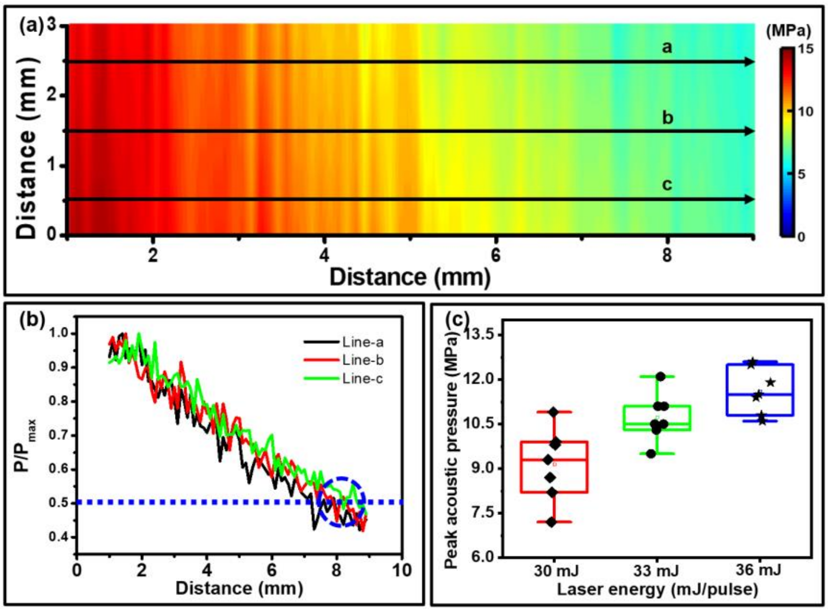

Fig. S18 Approximate experimental results. **a** Variation of optoacoustic pressure at laser intensity of 23 mJ/pulse. **b** Sound pressure with different distances in the three lines. **c** Variation of sound pressure at various laser energy at a distance of 8 mm

As shown in **Fig. S18a**, the output sound pressure attenuates remarkably as the distance increases. When the distance is about 8 mm, the sound pressure decreases to about half of the initial (**Fig. S18b**). Therefore, by testing the sound pressure value of 8 mm, the peak sound pressure output generated by the optoacoustic patch can be estimated. **Figure S18c** shows the

variation of sound pressure at a distance of 8 mm at different laser energy. Since the peak sound pressure is greater than 12.5 MPa at 36 mJ/pulse, it is inferred that the peak sound pressure of the patch can exceed 25 MPa.

Captions for Supplementary Movies

Movie S1 Acoustic flow experiment based on the optoacoustic patch (6.7 wt%, 23 mJ/pulse)

Movie S2 Control experiment: acoustic flow experiment without the optoacoustic patch (6.7 wt%, 23 mJ/pulse)

Movie S3 Thrombolysis experiment based on optoacoustic patch-thin blood clot (6.7 wt%, 23 mJ/pulse)

Movie S4 Thrombolysis experiment based on optoacoustic patch-thick blood clot (6.7 wt%, 23 mJ/pulse)

Movie S5 Wireless energy harvesting based on the optoacoustic patch in air (6.7 wt%, 23 mJ/pulse)

Movie S6 Lighting experiment of wireless energy harvesting based on the optoacoustic patch (a red LED, five 47 μF capacitors in series, 6.7 wt%, 23 mJ/pulse)

Movie S7 Laser-based wireless energy harvesting (23 mJ/pulse)

Movie S8 Wireless energy harvesting based on the optoacoustic patch in water (6.7 wt%, 23 mJ/pulse)

Supplementary References

[S1] J. Kang, D. Son, G. J. N. Wang, Y. Liu, J. Lopez et al., Tough and water-insensitive self-healing elastomer for robust electronic skin, Adv. Mater. **30**(13), 1706846 (2018), https://doi.org/10.1002/adma.201706846

[S2] S. Bode, L. Zedler, F. H. Schacher, B. Dietzek, M. Schmitt et al., Self-healing polymer coatings based on crosslinked metallosupramolecular copolymers, Adv. Mater. **25**(11), 1634-1638 (2013). https://doi.org/10.1002/adma.201203865

[S3] Y. Liu, T. Chou, Self-healing polymers based on thermally reversible Diels–Alder chemistry, Polym. Chem. **4**, 2194-2205 (2013). https://doi.org/10.1039/C2PY20957H

[S4] M. Liu, J. Zhong, Z. Li, J. Rong, K. Yang, et al., A high stiffness and self-healable polyurethane based on disulfide bonds and hydrogen bonding. Eur. Polym. J. **124**, 109475 (2020). https://doi.org/10.1016/j.eurpolymj.2020.109475

[S5] C. Sun, H. Jia, K. Lei, D. Zhu, Y. Gao, et al., Self-healing hydrogels with stimuli responsiveness based on acylhydrazone bonds, Polymer **160**, 246-253 (2019). https://doi.org/10.1016/j.polymer.2018.11.051

[S6] J. Dai, Z. Wang, Z. Wu, Z. Fang, S. heliu, et al., Shape Memory Polymer Constructed by π–π Stacking with Ultrafast Photoresponse and Self-Healing Performance, ACS Appl. Polym. Mater. **5**, 4 (2023). https://doi.org/10.1021/acsapm.2c02192

[S7] M. Rajczakowska, M. Szeląg, K. Habermehl-Cwirzen, H. Hedlund, A. Cwirzen, Autogenous self-healing of thermally damaged cement paste with carbon nanomaterials subjected to different environmental stimulators, J. Build. Eng. **72**, 106619 (2023). https://doi.org/10.1016/j.jobe.2023.106619

[S8] J. Xie, L. Gao, J. Hu, Q. Li, J. He, Self-healing of electrical damage in thermoset

polymers via anionic polymerization, J. Mater. Chem. C **8**, 6025-6033 (2020). https://doi.org/10.1039/C9TC06989E

[S9] W. Y. Chang, W. Huang, J. Kim, S. Li, X. Jiang, Candle soot nanoparticles-polydimethylsiloxane composites for laser ultrasound transducers, Appl. Phys. Lett. **107**(16), 161903 (2015). https://doi.org/10.1063/1.4934587

[S10] Y. Li, Z. Guo, G. Li, S. L. Chen, Miniature fiber-optic high-intensity focused ultrasound device using a candle soot nanoparticles-polydimethylsiloxane composites-coated photoacoustic lens, Opt. Express **26**(17), 21700-21711 (2018). https://doi.org/10.1364/OE.26.021700

[S11] M. Faraz, M. A. Abbasi, P. Sang, D. Son, H. W. Baac, Stretchable and robust candle-soot nanoparticle-polydimethylsiloxane composite films for laser-ultrasound transmitters, Micromachines **11**(7), 631 (2020). https://doi.org/10.3390/mi11070631

[S12] J. Li, X. Lan, S. Lei, J. Ou-Yang, X. Yang et al., Effects of carbon nanotube thermal conductivity on optoacoustic transducer performance, Carbon **145**, 112-118 (2019). https://doi.org/10.1016/j.carbon.2019.01.025

[S13] B. Y. Hsieh, J. Kim, J. Zhu, S. Li, X. Zhang et al., A laser ultrasound transducer using carbon nanofibers–polydimethylsiloxane composite thin film, Appl. Phys. Lett. **106**(2), 021902 (2015). https://doi.org/10.1063/1.4905659

[S14] J. Li, Y. Yang, Z. Chen, S. Lei, M. Shen et al., Self-healing: A new skill unlocked for ultrasound transducer, Nano Energy **68**, 104348 (2020). https://doi.org/10.1016/j.nanoen.2019.104348

[S15] T. Lee, L. J. Guo, Highly efficient photoacoustic conversion by facilitated heat transfer in ultrathin metal film sandwiched by polymer layers, Adv. Opt. Mater. **5**(2), 1600421 (2017). https://doi.org/10.1002/adom.201600421

[S16] M. Mohammadzadeh, S. R. Gonzalez-Avila, Y. C. Wan, X. Wang, H. Zheng, C. D. Ohl, Photoacoustic shock wave emission and cavitation from structured optical fiber tips, Appl. Phys. Lett. **108**(2), 024101 (2016). https://doi.org/10.1063/1.4939511

[S17] T. Lee, H. W. Baac, Q. Li, L. J. Guo, Efficient photoacoustic conversion in optical nanomaterials and composites, Adv. Opt. Mater. **6**(24), 1800491 (2018). https://doi.org/10.1002/adom.201800491

[S18] D. Kim, M. Ye, C. P. Grigoropoulos, Pulsed laser-induced ablation of absorbing liquids and acoustic-transient generation, Applied Physics A **67**, 169-181 (1998). https://doi.org/10.1007/s003390050756

[S19] G. J. Diebold, T. Sun, M. I. Khan, Photoacoustic monopole radiation in one, two, and three dimensions, Phys. Rev. Lett. **67**(24), 3384 (1991). https://doi.org/10.1103/PhysRevLett.67.3384

[S20] L. V. Wang, Tutorial on photoacoustic microscopy and computed tomography, IEEE J. Sel. Top. Quantum Electron. **14**(1), 171-179 (2008). https://doi.org/10.1109/JSTQE.2007.913398
